# Usefulness of hybrid deformable image registration algorithms in prostate radiation therapy

**DOI:** 10.1002/acm2.12515

**Published:** 2018-12-27

**Authors:** Kana Motegi, Hidenobu Tachibana, Atsushi Motegi, Kenji Hotta, Hiromi Baba, Tetsuo Akimoto

**Affiliations:** ^1^ Section of Radiation Safety and Quality Assurance National Cancer Center Hospital East Kashiwa‐shi Japan; ^2^ Particle Therapy Division Exploratory Oncology Research and Clinical Trial Center National Cancer Center Kashiwa‐shi Japan; ^3^ Division of Radiation Oncology National Cancer Center Hospital East Kashiwa‐shi Japan

## Abstract

To evaluate the accuracy of commercially available hybrid deformable image registration (DIR) algorithms when using planning CT (pCT) and daily cone‐beam computed tomography (CBCT) in radiation therapy for prostate cancer. The hybrid DIR algorithms in RayStation and MIM Maestro were evaluated. Contours of the prostate, bladder, rectum, and seminal vesicles (SVs) were used as region‐of‐interest (ROIs) to guide image deformation in the hybrid DIR and to compare the DIR accuracy. To evaluate robustness of the hybrid DIR for prostate cancer patients with organs with volume that vary on a daily basis, such as the bladder and rectum, the DIR algorithms were performed on ten pairs of CT volumes from ten patients who underwent prostate intensity‐modulated radiation therapy or volumetric modulated arc therapy. In a visual evaluation, MIM caused unrealistic image deformation in soft tissues, organs, and pelvic bones. The mean dice similarity coefficient (DSC) ranged from 0.46 to 0.90 for the prostate, bladder, rectum, and SVs; the SVs had the lowest DSC. Target registration error (TRE) at the centroid of the ROIs was about 2 mm for the prostate and bladder, and about 6 mm for the rectum and SVs. RayStation did not cause unrealistic image deformation, and could maintain the shape of pelvic bones in most cases. The mean DSC and TRE at the centroid of the ROIs were about 0.9 and within 5 mm generally. In both software programs, the use of ROIs to guide image deformation had the possibility to reduce any unrealistic image deformation and might be effective to keep the DIR physically reasonable. The pCT/CBCT DIR for the prostate cancer did not reduce the DIR accuracy because of the use of ROIs to guide the image deformation.

## 1 INTRODUCTION

The prostate is known to undergo motion and shape variations^1^, ^2^, ^3^, ^4^, ^5^, ^6^, ^7^, ^8^, ^9^ during a course of radiation therapy, caused by physiological changes, such as bladder volume changes and rectum filling. In image‐guided radiotherapy, the prostate is imaged using kilo‐voltage (kV) or mega‐voltage cone‐beam computed tomography (CBCT) to take into account the variety of the prostate position before the treatment.

To evaluate the daily shape and positional variations of the prostate during radiation therapy, deformable image registration (DIR), which is a non‐rigid image registration process to find corresponding points between CT volumes, is used to track complex organ motion and deformation on a voxel level. In contrast, a rigid image registration can estimate rotations and translations relative to one another by finding the most similar planes between CT volumes.

Deformable image registration is essential for the recently developed adaptive radiation therapy; exported deformation vector fields (DVFs), which represent the shift value and direction for a particular voxel to match to a corresponding voxel, are used for dose accumulation^10^ and automatic contour propagation.^11^ The DVFs exported from the DIR between planning CT (pCT) and daily CBCT are useful for evaluation of the prostate motion and deformation during a treatment. Moreover, the impact of organ motion and deformation, caused by variable volumes such as those of the bladder and rectum, on the prostate can be estimated using the DVFs.

However, it has been reported that DIR using the CBCT for the prostate region has poor accuracy because of unfavorable conditions such as noise, poor low‐contrast resolution, and abdominal motion artifacts.^12^ An intensity‐based DIR algorithm evaluates image similarity between CT volumes by using image intensities in an optimization problem. To measure the image intensity, mathematical methods such as the sum‐of‐squared differences, correlation coefficients, and mutual information are employed. Similar image intensities are found at many points in homogeneous regions and may cause unrealistic image transformation. Murphy^13^ investigated the influence of noise of the CBCT on the intensity‐based DIR accuracy by simulating the fan‐beam CT/CBCT registration in the presence of added target image noise. They found that DIR accuracy with a noisy CBCT was within a level consistent with inter‐observer variability for the purpose of automatic contouring. Furthermore, when CT volumes are registered deformably, every point must have a corresponding point in the other. The variable presence of bowel gas during and between treatment causes errors in the correspondence. Foskey^14^ processed each image exhibiting the problem to shrink the gassy region to a point using their “deflation” algorithm in the DIR.

A feature‐based DIR algorithm needs to define relevant landmarks such as points or contours in both the reference and target volumes. Because, landmarks in a target volume are transferred to match to the corresponding landmarks in the reference volume, human anatomy can be taken into account. The feature‐based method is useful for noisy CBCT images. However, the selection of landmarks is usually time‐consuming and similarity of the landmarks hides unrealistic image transformations in regions that are not of interest.^15^, ^16^

A hybrid DIR algorithm, which is based on the intensity‐based DIR algorithm combined with the feature‐based technique, has been reported by Kim et al.^17^ They employed their piecewise rigid registration method for femoral and pelvic bone registrations to preserve their shape. Mean contours and point features were then incorporated as constraints into a B‐spline‐based DIR algorithm. They reported high dice similarity coefficients (DSCs), which represent a spatial overlap of region‐of‐interest (ROIs) between CT volumes, of 0.9 for the prostate, rectum, and bladder, and 0.8 for seminal vesicles (SVs). Recently, the hybrid DIR algorithm has been commercially available in RayStation (RaySearch Laboratories AB, Stockholm, Sweden), MIM Maestro (MIM software Inc. Cleveland, OH, USA), and Velocity AI (Varian Medical Systems, Palo Alto, CA, USA). Weistrand and Svensson^18^ used the hybrid DIR algorithm in RayStation. A pair of volumes of pCT and CBCT from two prostate patients was tested, for whom they reported high DSCs of more than 0.9 for the prostate, rectum, and bladder. Thus, the hybrid DIR algorithm may be useful for pCT/CBCT DIR in the prostate region.

There have been few reports regarding the hybrid DIR algorithm for use in pCT/CBCT of the prostate region. In this study, we investigated the feasibility of a commercial hybrid DIR algorithm in pCT/CBCT in the prostate region. Volumes of pCT/CBCT from ten patients who underwent radiation therapy were used to evaluate robustness of the hybrid DIR for prostate cancer patients, using the DIR algorithms in RayStation (V. 4.7.4) and MIM Maestro (V. 6.6.8) software.

## 2 MATERIALS AND METHODS

### 2.A Patients

To evaluate the accuracy of the commercial hybrid DIR algorithms, ten prostate cancer patients who underwent intensity‐modulated radiation therapy or volumetric modulated arc therapy were selected. This retrospective study was reviewed by our institutional review board. All patients were prescribed a dose of 76 Gy in 38 fractions. They were instructed to empty their rectum and bladder, and subsequently drink 500 ml of water 30 min before the pCT and treatment.

### 2.B Datasets

A pair of the volumes of the pCT and the CBCT was respectively obtained by a CT‐simulation and a routine image acquisition before the treatment. The pCT images were acquired using an Aquilion One (Toshiba Medical Systems, Otawara, Japan). The matrix size was 512 × 512 pixels in the axial plane, and the pixel size was 0.94 mm × 0.94 mm. The field of view (FOV) was 480 mm in diameter. The images were reconstructed using 3 mm slice thickness. The CBCT images were acquired using an On‐Board Imager (Varian Medical Systems, Palo Alto, CA, USA). The scan mode selected was “pelvis spot light” with a full bow‐tie filter to improve the image resolution for the delineation. The imaging conditions were 125 kV, 80 mA, 25 ms, and with 270 projections during a half rotation covering a FOV diameter of 25 cm and a craniocaudal extension of 16 cm. The matrix size was 512 × 512 pixels in a plane, and the pixel size was 0.49 mm × 0.49 mm. The images were reconstructed using 1 mm slice thickness.

An experienced radiation oncologist delineated the prostate, rectum, bladder, and SVs on the pCT and CBCT images for all patients.

### 2.C Commercial deformable image registration software programs

RayStation employs the ANAtomically CONstrained Deformation Algorithm (ANACONDA) based on free‐form deformation (FFD) as the hybrid DIR algorithm. Details of the algorithm are described by Weistrand and Svensson and in other reports.^18^, ^19^, ^20^ The non‐linear optimization problem in ANACONDA is performed using the combination of four terms. Similarity between images is measured by a correlation coefficient in the first term of the problem. The second term of the problem is a regularization, which penalizes large shape deviations of ROIs. When an ROI type is set to “Avoidance” or “Organ”, the ROI is considered in the term. When a user includes controlling ROIs and points‐of‐interest to guide image deformation using key organs and points, the contour regularization term and a contour matching term are added in the third and fourth term of the problem, aimed at deforming the selected structure in the target image to the corresponding structure in the reference image.^19^ The terms allow fast convergence even for ROIs with large differences in size. This situation often occurs for bladders with different filling conditions.^18^ When FOCUS ROIs are selected, the ROIs are defined as focus regions of the DIR. Gaussian smoothing is applied in ANACONDA to prevent noisy deformation grids.

MIM employs the VoxAlign Deformation Engine for the DIR, which is a constrained, intensity‐based, FFD algorithm.^19^, ^20^, ^21^ Image similarity is measured using the sum‐of‐squared differences technique. Multiple types of regularization are used to keep the transformation reasonable. The algorithm actively attempts to match bone and avoid tears/folds in the deformation field.^22^

### 2.D Deformable image registration instructions

A pair of pCT and CBCT was respectively set as the reference and target volumes for the DIR. Since a rigid image registration must exist before initiating DIR in RayStation and MIM, pCT and CBCT were rigidly registered before the DIR. No mask algorithms to account for rectal and bowel gas were applied.

In the hybrid DIR for RayStation, the contours of the prostate, bladder, rectum, and SVs were used as the controlling ROIs to guide the image deformation. Although it is possible to ignore image information with controlling ROIs, the setting was not applied for the hybrid DIR using image intensity and geometrical information. Since MIM has no controlling ROIs for the hybrid DIR, the hybrid DIR using the image and geometrical information was performed using the flow of the geometrical‐based DIR. In the flow of the geometrical‐based DIR in MIM, images were registered deformably using image intensity first, and then MIM sets landmarks automatically on the surface of the selected ROIs for prostate, bladder, rectum, and SVs. The images are further transformed to match the ROIs to one another. Thus, the geometrical‐based DIR was classified to the hybrid DIR. The MIM software includes the Reg Reveal and Reg Refine tools, which allow users to view and refine deformation locally. To find the fundamental accuracy of the DIR, these tools were not used in this study. For the comparison, the intensity‐based DIR was performed in both software.

In DIR algorithm for RayStation, the initial and final grid resolution size was 5 mm isotropic and 2.5 mm × 2.5 mm × 3 mm in the right‐left, posterior‐anterior, and inferior‐superior (IS) directions. The grid size for the IS direction was similar to the slice thickness of the pCT and not user adjustable. Initial and final Gaussian smoothing sigma were 2 and 0.33. Initial and final grid regularization weight was 400. Maximum number of iteration per resolution level was 1000. An inverted grid was checked to avoid any irregular deformation. When the hybrid DIR was performed, the controlling ROI weight was 0.5. In MIM, the resolution grid size and weight are not user adjustable. The final grid resolution has a maximum grid size of 3 mm × 3 mm × 3 mm as of v6.4.5. Smoothing coefficient was 0.5. Because the CBCT could not include the full body in the small FOV, the focus‐region of the DIR was set to the imaged area of the CBCT in MIM and RayStation. FOCUS ROI was used in RayStation.

### 2.E Evaluation

The DIR accuracy was evaluated visually from the aspects of the unrealistic image deformation of soft tissue, organs, and pelvis bones.

Quantitative evaluation of the DIR accuracy was performed using DSC for the ROIs, target registration error (TRE) at the centroid of the ROIs and multiple evaluation points, and Jacobian determinants (JD) of the ROIs according to the American Association of Physicists in Medicine (AAPM) publication Task Group No 132 (TG‐132).^23^ Volume agreement between the pCT and deformed CBCT was expressed using the DSC:

(1) $$DSC=\frac{2\cdot \left|V_{r}\cap V_{d}\right|}{\left|V_{r}\right|+\left|V_{d}\right|}.$$

in eq. (1) represents the volume. A DICE score of 1 refers to two organs that overlap perfectly whilst 0 is of two ROIs that do not overlap at all.

The spatial discrepancy at the centroid position of the ROIs between pCT and deformed CBCT was expressed by the TRE and calculated by:

(2) $$TRE=\sqrt[{}]{{\left(x_{r}-x_{d}\right)}^{2}+{\left(y_{r}-y_{d}\right)}^{2}+{\left(z_{r}-z_{d}\right)}^{2}}.$$

and “r” and “d” in eqs. (1) and (2) refer to the pCT of the reference and deformed CBCT, respectively. Moreover, for the quantifiable accuracy of the non‐structure deformation, ten identifiable landmarks away from the contours of ROIs were evaluated using TRE.

To identify the local volume change as a result of the registration, JD were calculated using the script in RayStation and the statistical tool in MIM software. JD greater than 1 indicate volume expansion, between 0 and 1 indicate volume reduction, a value of 1 indicates no change, and a value of less than or equal to 0 indicates nonphysical motion. A Jacobian determinant of less than or equal to zero clearly indicates an erroneous physical modeling of the patient and may indicate an error in the registration or a limitation in the algorithm to handle complex deformation.^23^

To assess the statistical significance of the mean DSC, TRE, and JD between the intensity‐based DIR and hybrid DIR, *P*‐values by two‐tailed paired t‐test were evaluated. The t‐tests were performed between the DIRs of each software packages.

## 3 RESULTS

### 3.A Visual evaluation

**Figure 1:**
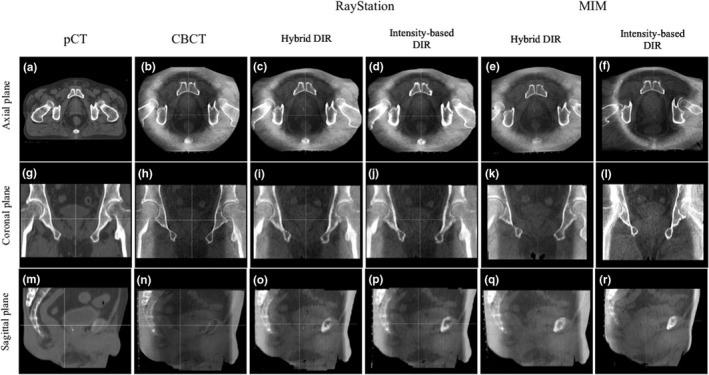
Comparison of the deformed CT images between the hybrid and intensity‐based deformable image registration (DIR) in Patient 1. The left two columns show the planning CT and the cone‐beam computed tomography images as a reference and a target for the DIR. The other four columns show images deformed by the hybrid and intensity‐based DIR algorithms. Patient 1 was a good case to understand the usefulness of the hybrid DIR in both commercial algorithms.

Figure 1 shows a comparison of the intensity‐based DIR and hybrid DIR for Patient 1 in both software.

In the DIR for RayStation, expansion of soft tissue at the image edges was observed in Fig. 1(c) and 1(d). The bladder for the patient had a volume change about 30 cc from the pCT to CBCT. When using the hybrid DIR, the agreement of the bladder improved as seen in Fig. 1(o). In the intensity‐based DIR, two of the cases had unrealistic image deformation of the external body shape. However, this was corrected in the hybrid DIR.

In the DIR for MIM, when using the intensity‐based DIR, soft tissue was expanded at the image edges as seen in Fig. 1(f) and eight cases had physically unrealistic deformation of bone structure. The expansion of the image edge was caused by the small FOV for the CBCT. The intensity‐based DIR tended to move the rectum and prostate toward their buttocks. When using the hybrid DIR, four cases had less physically unrealistic deformation of bone structure and seven cases reduced the soft tissue expansion at the image edges as seen in Fig. 1(e). Physically unrealistic deformation did not improve in two cases with the hybrid DIR.

### 3.B Quantitative evaluation

#### 3.B.1 Volume agreement

**Figure 2:**
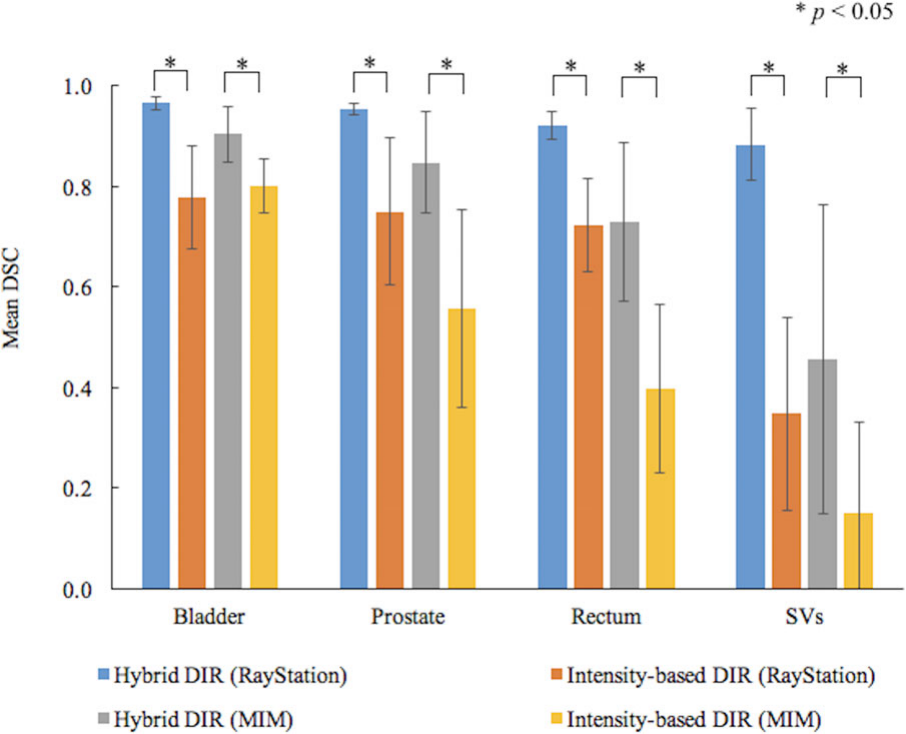
Comparison of mean dice similarity coefficient for the ROIs between the hybrid and intensity‐based DIR. Error bars show one standard deviation for the data from ten patients. When the significant differences were detected between the hybrid and intensity‐based DIR by t‐test, *P*‐values were indicated in the figure.

Figure 2 shows the mean DSCs from MIM and RayStation, with the error bars showing one standard deviation (SD).

In RayStation, the hybrid DIR had higher DSCs in all ROIs than the intensity‐based DIR. The mean DSCs for each ROI ranged 0.88‐0.97 with small SDs of about 0.03. In the intensity‐based DIR, the DSCs decreased by 0.23 with the DSC variations increasing by 0.08. The DSC for SVs decreased by 0.5.

In MIM, the hybrid DIR had higher DSCs in all ROIs than the intensity‐based DIR. The mean DSCs for each ROI ranged 0.46‐0.90 with large SDs of about 0.16. In the intensity‐based DIR, on average, the mean DSCs for each ROI decreased by 0.25 with SDs of 0.15. The lowest DSC of 0 was found for the SVs in patient 5. The statistical differences were obtained between the DIRs in each software package.

#### 3.B.2 Target registration error

**Figure 3:**
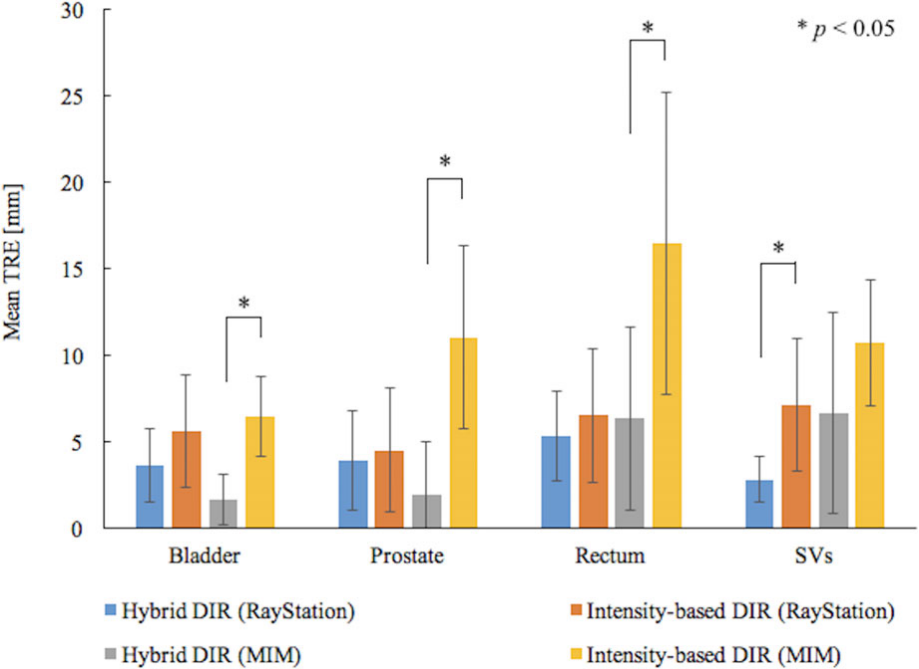
Comparison of mean Target registration error (TRE) at the region‐of‐interests (ROIs) between the hybrid and intensity‐based DIR. Error bars show one standard deviation for the data from ten patients. When the significant differences were detected between the hybrid and intensity‐based DIR by t‐test, *P*‐values were indicated in the figure.

**Figure 4:**
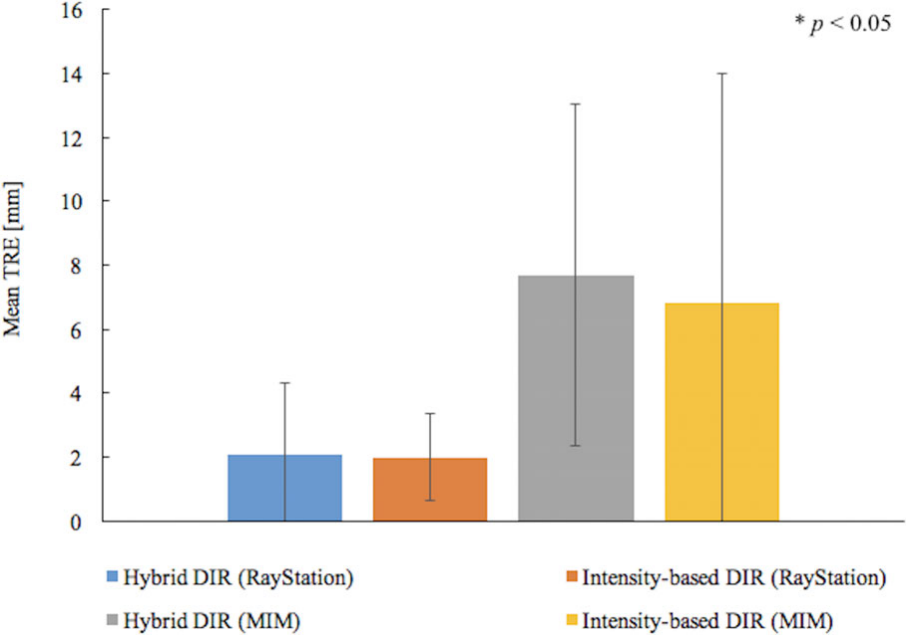
Comparison of mean TRE for the multiple evaluation points between the hybrid and intensity‐based DIR. Error bars show one standard deviation for the data from ten evaluation pointes for ten patients. When the significant differences were detected between the hybrid and intensity‐based DIR by t‐test, *P*‐values were indicated in the figure.

Figures 3 and 4 show the mean TRE at the centroid of the ROIs and multiple evaluation points between the pCT and the deformed CBCT. Error bars were added in the figure to show one SD of the TRE.

In RayStation, the mean TREs at the centroid of the ROIs, on average, were reduced from about 6 to 4 mm and the SD also decreased by about 1 mm when using the hybrid DIR. In the t‐test, the statistical differences were found in the SVs. For multiple evaluation points, the average TREs were about 2 mm in both DIRs and smaller SD were found in the intensity‐based DIR. No significant difference was obtained between the DIRs.

In MIM, the intensity‐based DIR had the mean TRE for each ROI ranged about 6‐17 mm. The maximum TRE of 35 mm was found. When using the hybrid DIR, MIM could reduce the TRE by 7 mm on average. In the t‐test of the TREs, the statistical differences were found between the DIRs, except for the SVs. For multiple evaluation points, the average TREs for both DIRs were more than 6 mm and average SD were more than 5 mm. No significant difference was found between the DIRs.

#### 3.B.3 Local volume change

**Figure 5:**
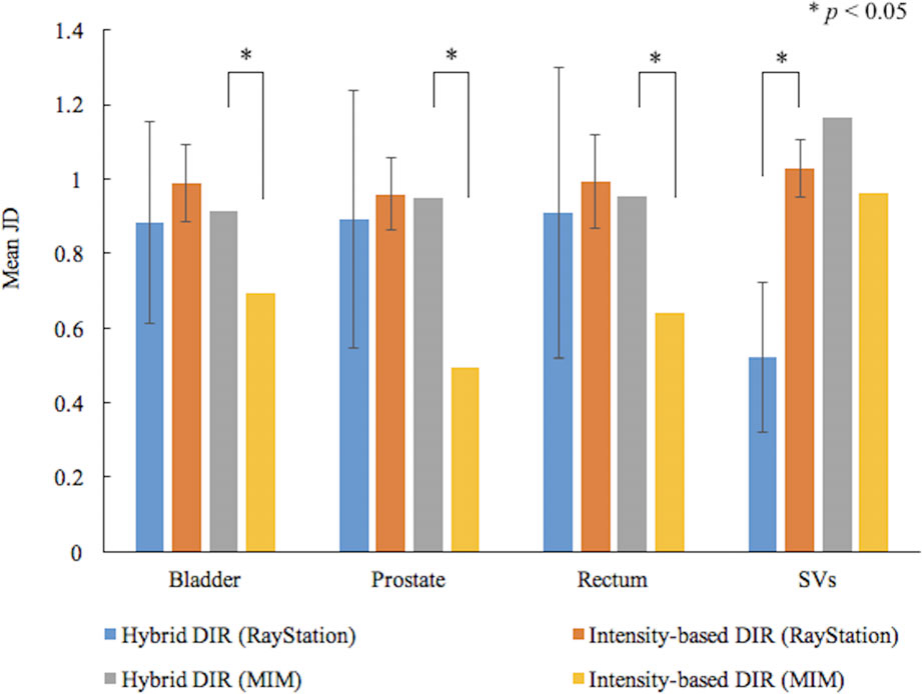
Comparison of mean Jacobian determinants for the ROIs between the hybrid and intensity‐based DIR. Error bars show one standard deviation for the data from ten patients. When the significant differences were detected between the hybrid and intensity‐based DIR by t‐test, *P*‐values were indicated in the figure.

Figure 5 shows the mean JD for the ROIs with the error bars showing one SD between the pCT and the deformed CBCT. Since the SD for MIM was not obtained using the statistical tool, no error bars were added.

In RayStation, the mean JD for the bladder, prostate, and rectum were reduced from about 1.0 to 0.9 when using the hybrid DIR. The JD for SVs degreased significantly by about 0.5. The SD, on average, increased by 0.2 in the hybrid DIR.

In MIM, the mean JD ranged from about 0.5 to 1.0 and the volume reduction was detected expect for the SVs in the intensity‐based DIR. Some cases had the value less than 0. When using the hybrid DIR, the mean JD, on average, increased by about 0.3 and the volume reduction significantly improved.

## 4 DISCUSSION

In MIM, nonphysical deformation of soft tissues, organs, and pelvic bones was observed in the intensity‐based DIR. Even if the ROIs of the prostate, bladder, rectum, and SVs to guide the image deformation were applied to the hybrid DIR, the correspondence between the pCT and the deformed CBCT was incomplete and deformation of the bone shape remained in half of the cases. The failure cases had SVs that were closely surrounded by the prostate, bladder, and rectum. In those cases, the hybrid DIR might have caused irregular image deformations. When ROIs have no room to deform, the DIR might fail.

In AAPM TG‐132, the TRE tolerance should be no more than maximum volume dimension so 3 mm and DSC tolerance should be from 0.8 to 0.9. Since the DSC value varies dependent on structure size such as SVs with small volume, the lowest DSC for SVs was reasonable. The JD is evaluated according to the clinical scenario. In the pCT/CBCT DIR for the prostate radiation therapy, the JD for the prostate and SVs are expected 1 and variable for the rectum and bladder.

In the DIR algorithm for MIM, even though the hybrid DIR improved the DSC and TRE at the centroid of the ROIs, the values did not clear the tolerance. The TRE for the multiple evaluation points, which shows the accuracy of the non‐structure deformation, had no improvement. The JD for the prostate were close to the expected value and the volume for the SVs was overexpanded.

Limitations of the algorithm including registering noisy CT images, image pairs with inconsistent Hounsfield Units, and image pairs with regions for which no correspondence exists, such as fecal matter and gas in the rectum, were mentioned in the MIM User Guide Version 6.5. In general, the entire external outline of body should be included in image pairs for reliable results at the image edge. The DIR between the pCT and the CBCT in this study did not satisfy the conditions. With some additional fine‐tuning such as the Reg Reveal and Reg Refine tools, the MIM would generate better results. For the clinical use, users should consider the limitation of the hybrid DIR.

In the DIR algorithm for RayStation, no unrealistic image deformation of the soft tissues, organs, and pelvic bones was observed in most cases. Because RayStation implemented shape‐based grid regularization in the optimization problem for the anatomically reasonable deformation, the DIR algorithm in RayStation could be robust for image pairs with poor image quality caused by noise and artifacts. For the expansion of soft tissue at the image edges, outside the FOCUS ROI the deformation is smoothed to vectors of 0 over a small range, resulting in allowed small deformations just outside the focus. This might result in some of the errors at the edge of the images.

In the hybrid DIR in RayStation, controlling ROIs considered in the penalty term of the optimization problem improved the correspondence of the bladders and rectums with variable volume and their DSC values up to 0.97. The mean TRE at the centroid of the ROIs was also reduced to less than 5 mm in eight cases. The DSC for ROIs and the mean TRE for the multiple evaluation points cleared the tolerance indicated in TG‐132. Thus, the hybrid DIR was useful for the pCT/CBCT DIR in the prostate radiation therapy.

However, in the hybrid DIR in RayStation, the deformation using the geometrical information showed almost complete matching between the pCT and deformed CBCT. This means the DIR algorithms forced the ROIs to agree strongly. Thus, users should be careful to delineate contours used for the controlling ROIs. The DIR accuracy is affected directly by the accuracy of the delineation. The inter‐observer variability for the delineation of the prostate and SVs has been reported 1 cm in IS direction among five well‐trained radiotherapists and the percentage SD of the ROI volumes ranged from 10% to 18%.^24^ Moreover, for a routine clinical implementation of the DIR, delineating contours for every CBCT images is time‐consuming and overloaded. The use of auto‐segmentation tools on the hybrid DIR would be interesting in future work.

For a fair comparison, the grid resolution for the DIR should have been the same in RayStation and MIM. RayStation had the smaller grid resolution compared to MIM in this study. From this aspect, RayStation was thought to deliberately bringing better results. The grid resolution for both software was not user adjustable with arbitrary value. Users can select the grid size from 1 mm × 1 mm × 3 mm, 2.5 mm × 2.5 mm × 3 mm, and 5 mm × 5 mm × 5 mm in RayStation. In order to investigate the influence of the grid resolution on the DIR accuracy in RayStation, comparisons in different grid resolution were performed. As shown in Table 1, the DIR accuracy with the largest grid resolution of 5 mm × 5 mm × 5 mm had the least TRE. No significant difference in the comparison of DSC and JD was detected. Therefore, there is little possibility that the grid resolution in RayStation affects the result of the comparison of the two software in this study.

**Table 1:** Comparison of the DIR accuracy in different grid resolution in RayStation. The DSC and JD for the ROIs and TRE for the multiple points were calculated using the analysis tools in the software. The average values for ten patients were tabulated. The significant difference was detected by two‐tailed paired t‐test (*p *<* *0.05)

Grid resolution (mm)	Hybrid DIR			Intensity‐based DIR		
	1 × 1 × 1	2.5 × 2.5 × 3	5 × 5 × 5	1 × 1 × 1	2.5 × 2.5 × 3	5 × 5 × 5
DSC						
Bladder	0.98 ± 0.01	0.97 ± 0.01	0.96 ± 0.01^a^	0.77 ± 0.10	0.78 ± 0.10	0.77 ± 0.10
Prostate	0.96 ± 0.01	0.96 ± 0.01	0.95 ± 0.01	0.74 ± 0.14	0.75 ± 0.14	0.74 ± 0.14
Rectum	0.93 ± 0.02	0.93 ± 0.03	0.91 ± 0.02	0.70 ± 0.09	0.72 ± 0.09	0.70 ± 0.08
SV	0.87 ± 0.09	0.88 ± 0.08	085 ± 0.08	0.35 ± 0.21	0.38 ± 0.20	0.35 ± 0.21
JD						
Bladder	0.90 ± 0.27	0.91 ± 0.26	0.91 ± 0.26	0.98 ± 0.02	1.00 ± 0.03	1.00 ± 0.01
Prostate	0.87 ± 0.27	0.87 ± 0.27	0.87 ± 0.26	0.96 ± 0.04	0.97 ± 0.05	1.00 ± 0.01
Rectum	0.93 ± 0.23	0.93 ± 0.23	0.91 ± 0.22	0.93 ± 0.06^a^	0.99 ± 0.05	0.99 ± 0.01
SV	0.49 ± 0.21	0.48 ± 0.22	0.48 ± 0.20	0.97 ± 0.04^a^	1.03 ± 0.07	1.00 ± 0.02
TRE (mm)						
	2.2 ± 1.8	2.4 ± 1.8	1.7 ± 1.7^a^	1.8 ± 1.2^a^	2.2 ± 1.3	1.4 ± 1.1^a^

DIR, deformable image registration; JD, Jacobian determinants; SV, seminal vesicles; DSC, dice similarity coefficient.

a *p *<* *0.05.

## 5 CONCLUSIONS

In this study, hybrid DIR algorithms in the RayStation and MIM Maestro were evaluated for the pCT/CBCT DIR in the prostate region when using non‐rigid image registration. The use of ROIs of prostate, bladder, rectum, and SVs to guide the image deformation had the possibility to reduce nonphysical image deformation and the DIR accuracy improved. However, the accuracy of the hybrid DIR algorithms had large variation between the two software packages. The hybrid DIR in RayStation was robust for the chosen prostate cancer patients.

## CONFLICT OF INTEREST

The authors declare no conflict of interest
